# Sex and Tissue Specificity of *Peg3* Promoters

**DOI:** 10.1371/journal.pone.0164158

**Published:** 2016-10-06

**Authors:** Bambarendage P. U. Perera, Joomyeong Kim

**Affiliations:** Department of Biological Sciences, Louisiana State University, Baton Rouge, LA, United States of America; University of Texas at Austin Dell Medical School, UNITED STATES

## Abstract

The expression of mouse *Peg3* (Paternally expressed gene 3) is driven by 4 promoters, including its main and three alternative promoters. The sexual, temporal and spatial specificity of these promoters was characterized in the current study. According to the results, the main promoter displays ubiquitous expression patterns throughout different stages and tissues. In contrast, the expression of *Peg3* driven by the alternative promoter U2 was detected mainly in muscle and skin, but not in brain, starting from the late embryonic stage, revealing its tissue and stage specificity. The expression levels of both the main and U2 promoters are also sexually biased: the levels in females start higher but become lower than those in males during early postnatal stages. As an imprinted locus, the paternal alleles of these promoters are active whereas the maternal alleles are silent. Interestingly, deletion of the repressed maternal allele of the main promoter has an unusual effect on the opposite paternal allele, causing the up-regulation of both the main and U2 promoters. Overall, the promoters of *Peg3* derive sexually biased and tissue-specific expression patterns.

## Introduction

*Peg3* (Paternally expressed gene 3) is an imprinted gene that is localized in human chromosome 19q13.4/mouse proximal chromosome 7 [1]. This gene is the founding member of an evolutionarily conserved 500-kb imprinted domain, which includes paternally expressed *Usp29*, *APeg3*, *Zfp264* and maternally expressed *Zim1*, *Zim2*, *Zim3* [2]. The 4-kb genomic region surrounding the bi-directional promoter for *Peg3* and *Usp29* is methylated during oogenesis, thus both *Peg3* and *Usp29* are expressed mainly from the paternal allele [3,4]. As an Imprinting Control Region (ICR), this 4-kb region has been shown to control the imprinting and transcription of the entire 500-kb domain [5]. In terms of *in vivo* functions, *Peg3* is the best studied so far among the genes in this domain. Mutagenesis experiments demonstrated that *Peg3* is likely involved in controlling fetal growth rates and also maternal-caring behaviors [6–8]. Consistent with this, the expression levels of *Peg3* are very high in embryos, placentas and brains [1,9,10]. Interestingly, the mutations tend to have more severe effects on males than on females, suggesting the presence of potential sexual bias associated with the *Peg3* locus [5,7,11,12]. This further suggests that the *Peg3* locus may be subject to some unknown regulatory mechanisms other than genomic imprinting [12].

The expression of mouse *Peg3* is driven by at least 4 individual promoters, including the known main promoter and three recently discovered alternative promoters, named U1 through U3 [13]. The alternative promoters, U1, U2, and U3, are localized 20, 26 and 163-kb upstream of the main promoter. The 250-kb genomic interval upstream of the main promoter is also filled with many Evolutionarily Conserved Regions (ECRs), which are putative transcriptional enhancers based on the close association with histone marks, such as H3K27ac (acetylation on lysine 27 of histone 3) and H3K4me1 (mono-methylation on lysine 4 of histone 3) [14,15]. This genomic juxtaposition of the alternative promoters with the potential enhancers suggests that the *Peg3* locus may be controlled through the long-range interactions between these *cis*-regulatory elements. This has been further supported by the recent finding that several imprinted domains contain oocyte-specific alternative promoters that are localized upstream of their main promoters [16]. Interestingly, these alternative promoters are responsible for the establishment of oocyte-specific DNA methylation on the downstream main promoters [16,17]. In the *Peg3* locus, the alternative promoter U1 is oocyte-specific, thus predicted to play a similar role as seen in the other imprinted domains, such as *Gnas* and *Zac1* domains. On the other hand, the potential roles of the other alternative promoters are currently unknown.

As part of an ongoing effort, we sought to characterize the features of the alternative promoters of mouse *Peg3* in the current study. According to the results, the main and U2 promoters display different patterns of stage and tissue specificity. The main promoter of *Peg3* exhibits relatively ubiquitous patterns whereas the U2 promoter is very stage and tissue-specific. On the other hand, both promoters display sexually biased expression patterns during late embryonic stages. More detailed results are presented below.

## Results

### Temporal expression patterns of *Peg3* promoters

**Fig 1:**
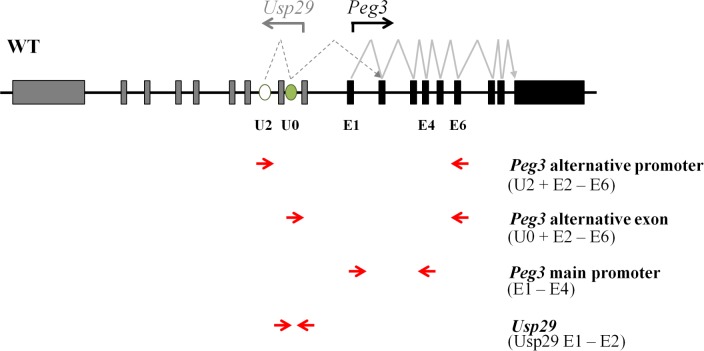
Schematic representation of the exon structures of mouse *Peg3* and *Usp29*. Gray and black boxes indicate the exons of *Usp29* and *Peg3*, respectively. The transcriptional direction for each gene is represented with an arrow. The open and closed ovals represent the alternative 1^st^ exon U2 and an additional exon U0, respectively. The arrows below the map indicate the directions of primers: U2 or U0 was coupled with E6 (exon 6) to target the alternative promoter U2 for *Peg3*; E1 (exon 1) was coupled with E4 (exon 4) to target the main promoter of *Peg3*; and the Usp29 E1-E2 (exon 1 and 2) was used to target the promoter of *Usp29*.

To characterize the promoters of mouse *Peg3*, we analyzed the expression patterns of each transcript that is driven by its corresponding promoter (Fig 1). Among the three alternative promoters, the U2 promoter is the only one that derives the expression in neonatal stages as well as in various tissues [13]. Thus, the current study has focused mainly on the U2 promoter along with the main promoter of *Peg3* (Fig 1). The two primer sets, U2-E6 and U0-E6, were designed to detect the expression patterns of the U2 promoter. The primer set E1-E4 is designed to detect the expression patterns of the main promoter of *Peg3*. Since the main promoter of *Peg3* is also bi-directional, this series of analyses also included the primer set Usp29 E1-E2 that was designed to detect the expression patterns of *Usp29*. The expression levels of these transcripts were also normalized with those of two internal controls, *Gapdh* and *β-Actin*.

**Fig 2:**
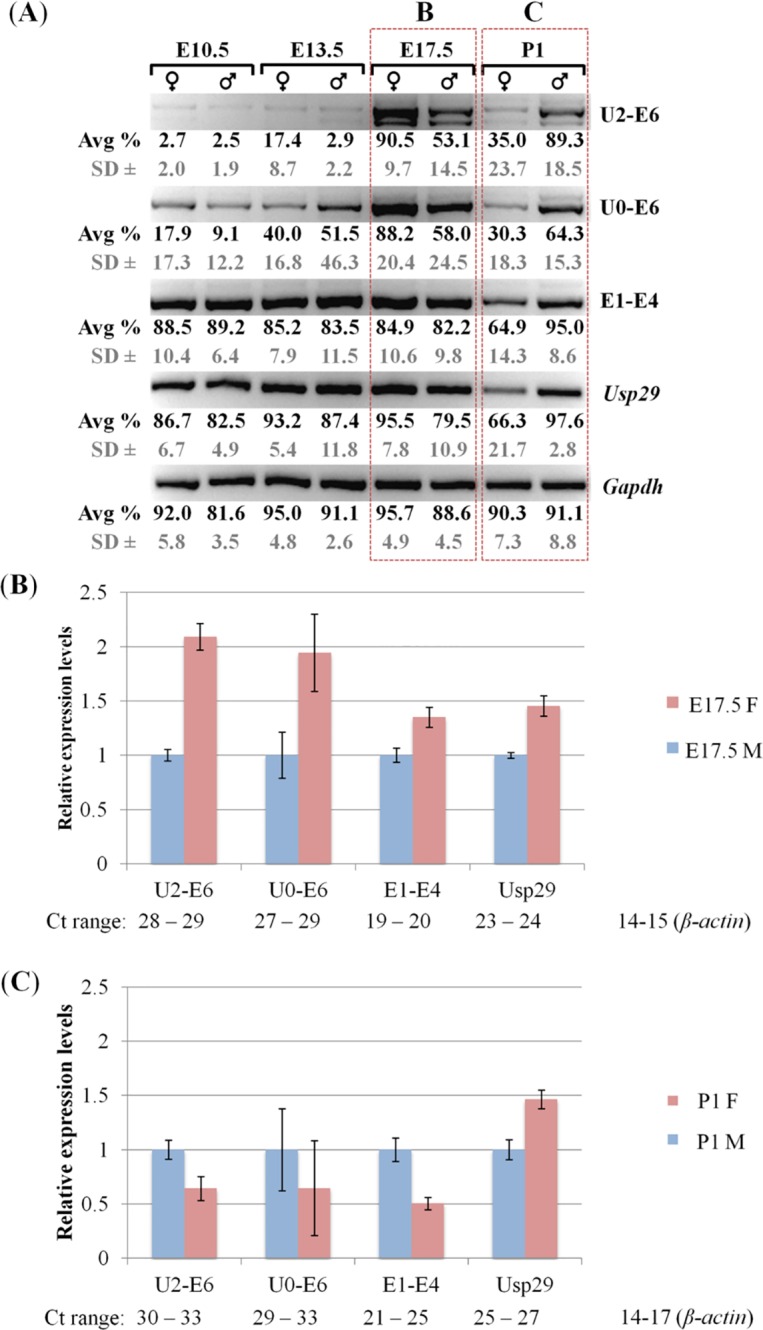
Sex and stage specificity of *Peg3* promoters. (A) The RT-PCR panel summarizes the expression patterns of the promoters of *Peg3* and *Usp29*. This series of analyses used a set of total RNA that had been isolated from the males and females of 10.5, 13.5, 17.5-dpc embryos and one-day-old neonates (P1). The primer set U2-E6 and U0-E6 were designed to detect the expression patterns of the alternative promoter of *Peg3*, while the primer set E1-E4 was to detect the expression patterns of the main promoter of *Peg3*. The primer set *Usp29* E1-E2 was designed to detect the expression patterns of the promoter of *Usp29*. The different amounts of individual cDNA were normalized with the expression levels of *Gapdh*. This series of analyses were repeated using three independent trials, where the average and the standard deviations of density for each band were measured as a percentage using the ImageJ32 program, as normalized values for each gel image. (B,C) Quantitative RT-PCR analysis using the total RNA isolated from the female (pink) and male (blue) set of E17.5 embryos and P1 neonates. The Ct values for each promoter was first normalized with an internal control (*β-actin*) and subsequently used for calculating the relative expression levels between females and males. The range of Ct values are also presented below the graph to indicate the relative expression levels of each amplicon. This series of analyses was repeated using two triplicate reactions for each sample with error bars indicating the standard deviations from a total of six replicates.

The temporal expression patterns of the promoters were first analyzed in the following manner. Several sets of female and male embryos representing different stages of embryogenesis, E10.5, E13.5, and E17.5, were harvested through timed mating of wild-type C57BL/6J mice. This series of analyses also included the one-day-old pups (P1). This series of analyses used the entire embryos of the following two stages, E10.5 and E13.5, but mainly the head portion of the pups of the remaining two stages, E17.5 and P1. The harvested tissues were used for isolating total RNA, which were then used for generating cDNA for two series of RT-PCR analyses. The first series was performed with RT-PCR involving fixed numbers of cycles (Fig 2A), whereas the second series was performed with quantitative RT-PCR (Fig 2B and 2C). The results from this series of expression analyses derived the following observations. First, the main promoter of *Peg3* (E1-E4) and the promoter of *Usp29* displayed ubiquitous expression patterns throughout the different embryonic stages, showing overall similar levels of the expression relative to those of the internal controls. Second, the U2 promoter (U2-E6) showed very low or undetectable levels of the expression at E10.5 and E13.5, but dramatically increased levels at E17.5 and P1. The levels at E17.5 were higher than those from P1. Similar patterns were also observed with the primer set U0-E6, but the observed levels by this primer set were more visible at the two early stages, E10.5 and E13.5, compared to U2-E6. Overall, the U2 promoter appeared to be very stage-specific with the highest levels peaking at E17.5. Third, both the main and U2 promoters of *Peg3* showed different levels of the expression between the two sexes: the relative levels of females to males were higher at E17.5 but lower at P1 (Fig 2B and 2C). The relative ratios of female to male were quite dramatic in the case of the U2 promoter, 2.1 at E17.5 (p = 0.0001) versus 0.6 at P1 (p = 0.0003). This was also the case for the main promoter, showing 1.4 at E17.5 (p = 0.0001) and 0.5 at P1 (p = 0.0001). However, the ratios observed from *Usp29* remained 1.5 throughout the two stages (p = 0.0001 at E17.5 and p = 0.0001 at P1). The sexually biased expression levels at the P1 stage were consistent with the previous observation that the expression levels of *Peg3* and other imprinted genes are higher in males compared to females [12]. On the other hand, the high levels in females at E17.5 has never been reported, and also the sexually biased expression levels with the opposite pattern between the E17.5 and P1 stages appeared to be very unusual. This series of expression analyses were repeated two times per each biological replicate and also with the two independent sets of biological replicates. Overall, this series of analyses uncovered the stage specificity of the U2 promoter as well as the sexually biased expression levels of both the main and U2 promoters of mouse *Peg3*.

### Spatial expression patterns of *Peg3* promoters

**Fig 3:**
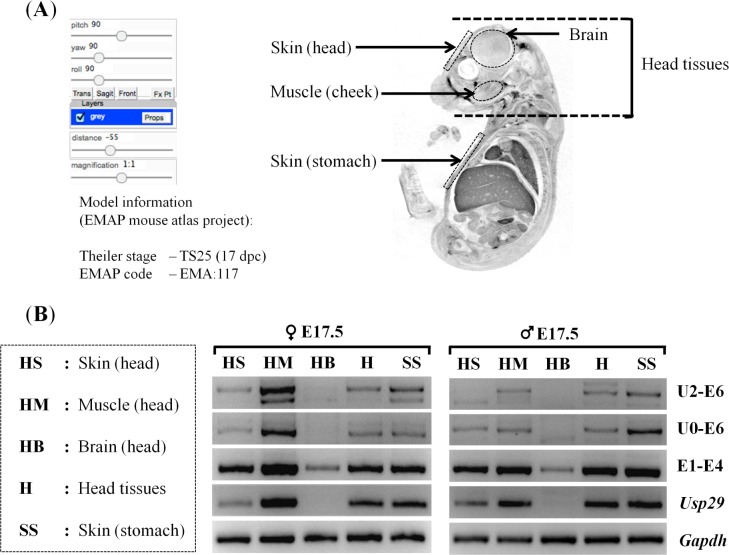
Spatial expression patterns of *Peg3* promoters. (A) The tissues used for RNA isolation were visualized using the image, which was derived from the EMAP mouse atlas project (http://www.emouseatlas.org), code EMA: 117 to indicate the proper anatomy of a 17-dpc embryo of a sagittal section [28]. The dotted areas indicate the regions where total RNA was isolated, including head skin (HS), cheek muscles (HM), brain (HB), the entire head tissues (H), and stomach skin (SS). (B) Spatial expression patterns of the *Peg3* promoters. The two RT-PCR panels show the tissue-specific expression patterns observed from the female (left) and male (right) of E17.5 embryos. The primer set U2-E6 and U0-E6 were designed to survey the expression patterns of the U2 promoter of *Peg3*, while the primer set E1-E4 and Usp29 were designed to survey the expression profile of the main promoter of *Peg3* and *Usp29*, respectively. The different amounts of cDNA between samples were normalized with the expression levels of *Gapdh*. The series of analyses were repeated three independent trials.

The tissue specificity of the promoters was also analyzed using the samples derived from different parts of embryos as well as neonatal and adult tissues (Fig 3 and S1 Fig). Total RNA was first isolated from the five different parts of E17.5 embryos of males and females, including the skin (HS), cheek muscle (HM), and brain (HB) of the embryo heads, the entire heads (H), and finally the skin from the stomach area (SS) (Fig 3A). The isolated RNA was subsequently used for generating a panel of cDNA, which was then used for a series of RT-PCR with fixed number of cycles (Fig 3B). This survey derived the following conclusions. First, the expression of both the main promoter of *Peg3* and *Usp29* was detected throughout all the different parts examined so far. However, the expression levels in the brain (HB) by both the main and *Usp29* promoters were relatively low compared to the levels from the other parts, as judged by their high ΔCt values (Fig 3B and S1A Fig). In particular, the levels of *Usp29* were undetectable in the brain (HB). On the other hand, the expression levels in the muscle (HM) were the highest among the tissues examined so far, showing very low ΔCt values (Fig 3B and S1A Fig). Second, similar patterns were also observed in the case of the U2 promoter: the expression levels in the brain (HB) were very low or undetectable with both primer sets, U2-E6 and U0-E6. In contrast, the expression levels were the highest in the muscle (HM). The observed expression patterns of the U2 promoter were also maintained until the P1 stage, but not as prominent in the adult (S1B Fig). On the other hand, the main promoter and the *Usp29* promoter become more brain-specific at the adult stages (S1B Fig). This series of expression analyses were repeated at least twice using a set of E17.5 embryos and two neonates, and the results were overall consistent with those presented above. Taken together, this series of analyses concluded that the U2 promoter is specific in non-neuronal cells, such as muscle and skin, whereas the main promoter is more ubiquitous.

### Peg3-ICR influence on the U2 promoter

**Fig 4:**
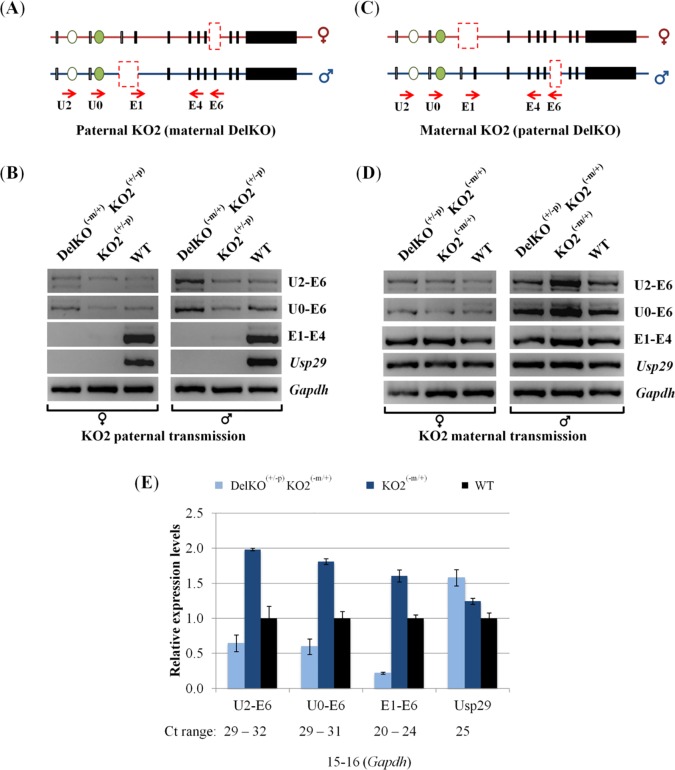
Deletion effects of the 4-kb Peg3-ICR on the U2 promoter. (A,C) A reciprocal cross of mouse breeding experiments were performed to derive two types of the pups with the paternal and maternal transmission of KO2 and DelKO (A) and the pups with the opposite transmission of the two alleles (C). The solid boxes indicate the position of the exons of *Peg3* and *Usp29*, while the ovals represent the upstream alternative exons. The dashed boxes indicate the deleted regions, exon 6 for DelKO and the 4-kb ICR containing the bidirectional promoter for *Peg3* and *Usp29* for KO2. (B,D) The pups with three different types were used for RNA isolation and subsequent RT-PCR analyses, including wild-type (WT), single heterozygotes for KO2 (KO2^+/-p^ or KO2^-m/+^), and double heterozygotes (DelKO^-m/+^ KO2^+/-p^ or DelKO^+/-p^ KO2^-m/+^). The expression levels of each promoter were compared among the pups with these genotypes. The different amounts of cDNA between samples were first normalized with the expression levels of *Gapdh*, and the normalized cDNA were compared among the three samples per each promoter. The primer set U2-U6 and U0-U6 were to detect the expression levels of the U2 alternative promoter, while the primer set E1-E4 and Usp29 were to detect the expression levels of the main promoter of *Peg3* and the promoter of *Usp29*, respectively. (E) Expression level analyses using qRT-PCR. This set of cDNA was same as the male set shown in Fig 5D. The Ct values for each promoter was first normalized with an internal control (*β-actin*) and subsequently used for calculating the relative expression levels compared to the levels of WT. The range of Ct values are also shown below the graph to indicate the relative expression levels of each amplicon. This series of analyses was repeated using triplicate reactions for each sample with error bars indicating the standard deviations from the triplicates. The data was consistent with at least two independent qRT-PCR experiments as shown by S3 Fig.

The 4-kb genomic region surrounding the bi-directional promoter of *Peg3* and *Usp29* is known to control the entire 500-kb imprinted domain [2,5]. Thus, we sought to test potential effects of the deletion of this ICR on the activity of the U2 promoter *in vivo*. For this series of analyses, a reciprocal cross of mouse breeding experiments was performed with the two mutant alleles targeting the *Peg3* locus (Fig 4A and 4C). The first mutant allele lacks the exon 6 of *Peg3*, named DelKO [13]. The two parental alleles could be easily differentiated through a RT-PCR scheme targeting the deleted region, the exon 6 (E6 primer). The second mutant allele lacks the entire 4-kb bi-directional promoter, named KO2 [13]. In this case, the mutants carrying this allele paternally do not express the transcripts driven by the bi-directional promoter of *Peg3* and *Usp29*. Males and females carrying these two mutant alleles were bred together, and the resulting pups were genotyped accordingly (S2B Fig). The heads of these neonates with different genotypes were used for isolating total RNA, which were then used for generating two panels of cDNA: the first set with the maternal and paternal transmission of DelKO and KO2, respectively, whereas the second set with the opposite transmission of these two alleles relative to the first set (Fig 4B and 4D). The breeding results indicated that the pups with all four genotypes are viable with a mendelian ratio, and is consistent with the previously observed phenotypes for each transmission (S2B Fig) [13].

The results from this series of analyses derived the following observations. First, it is inconclusive whether the paternal transmission of KO2 causes any major impact on the expression levels of the U2 promoter given the very low levels of expression of the U2 transcripts, although there might be no major impact based on no major difference between KO2^(+/-p)^ and WT samples (2^nd^ and 3^rd^ lane on Fig 4B). The transcript by the U2 promoter detected from the DelKO^(-m/+)^KO2^(+/-p)^ sample was derived from the paternal allele since both primer sets, U2-E6 and U0-E6, target only the remaining paternal allele of the exon 6 in this cross (1^st^ lane on Fig 4A). Yet, the expression levels of the transcript by the U2 promoter were similar between the samples with DelKO^(-m/+)^KO2^(+/-p)^ and KO^(+/-p)^ genotypes (1^st^ and 2^nd^ lane on Fig 4B). This indicated that the observed levels of the U2 promoter were mainly contributed by the paternal allele. Second, the maternal transmission of KO2 resulted in an unusual up-regulation in the expression levels of the main promoter as well as the U2 promoter (Fig 4D). The levels observed from the KO2^(-m/+)^ sample were greater than those from WT sample (2^nd^ and 3^rd^ lane on Fig 4D), and this up-regulation was detected in the U2 promoter as well as the main promoter of *Peg3* (1^st^ through 3^rd^ row on Fig 4D). This was also more obvious in the male set than in the female set due to the higher levels of the expression observed from the male set at the P1 stage (right panel on Fig 4D). On a separate note, the majority of the U2 transcript was likely derived from the paternal allele given the dramatic difference in the expression levels between the two samples with DelKO^(+/-p)^KO2^(-m/+)^ and KO^(-m/+)^ genotypes (1^st^ and 2^nd^ lane on the right panel of Fig 4D). To further confirm this, we also performed an independent series of qRT-PCR using a male set of cDNA with the maternal transmission of KO2 (Fig 4E and S3 Fig). As expected, the levels of the transcripts contributed by the paternal allele were indeed 3 to 7 times higher than those by the maternal allele given the expression level differences between the two samples with the DelKO^(+/-p)^KO2^(-m/+)^ and KO^(-m/+)^ genotypes (p = 0.0006 for the U2 promoter and p = 0.0001 for the main promoter, respectively). On the other hand, the expression levels driven by the U2 and main promoters were indeed twice and 1.6 times higher, respectively, in the KO2^(-m/+)^ sample than those in the WT sample, reconfirming the up-regulation by the maternal transmission of the KO2 allele (p = 0.0427 for the U2 promoter and p = 0.0001 for the main promoter). Thus, the maternal deletion of KO2 appeared to affect trans-allelically the paternal allele of both the main and U2 promoters of *Peg3*. Overall, this series of expression analyses concluded that the paternal deletion of the main promoter remains inconclusive in terms of the potential impact on the activity of the U2 promoter. However, the maternal deletion of the main promoter, as part of an ICR, resulted in an unusual up-regulation of the remaining paternal alleles of both the main promoter itself and the U2 promoter.

### DNA methylation analysis of *Peg3* promoters in neonates

**Fig 5:**
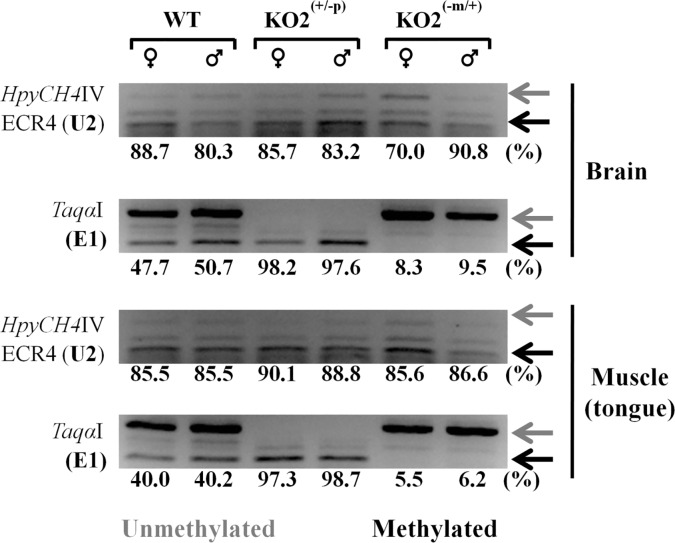
DNA methylation analyses of the promoters of *Peg3*. DNA was first isolated from the brain and muscle of the P1-stage female and male neonates, and these DNA were treated with the bisuflite conversion protocol. These converted DNA were amplified with the two primer sets targeting the alternative and main promoter of *Peg3*. The amplified PCR products were analyzed with COBRA involving restriction enzyme digestion. The restriction enzyme *HpyCH4*IV was used for the analysis of the U2 promoter, while the restriction enzyme *Taq*I was for the analysis of the main promoter of *Peg3*. The digested fragments in both cases represent the methylated status as indicated by black arrows, whereas the undigested DNA fragments represent the unmethylation status as indicated by grey arrows. The DNA methylation level of each sample was derived from the relative ratio of the digested versus undigested amounts of the DNA fragment. For this series of analyses, the density of each band was measured using the ImageJ32 program. The final methylation level of each sample was derived from the average value of three independent trials of COBRA.

The promoters of *Peg3* were also analyzed in terms of their DNA methylation levels (Fig 5). This series of analyses used the DNA isolated from the two different parts of the female and male neonates of the following genotypes: WT, KO2^(+/-p)^, and KO2^(-m/+)^. The DNA isolated from the brain and muscle of the one-day-old pups were treated with the bisulfite conversion protocol, and the converted DNA were subsequently used for PCR amplification targeting several regions, including the main and U2 promoters of *Peg3*. The amplified PCR products were analyzed with COBRA (Combined Bisulfite Restriction enzyme Analysis, [18]). The results from this series of analyses provided the following conclusions. First, the DNA methylation status of the main promoter was allele-specific as expected: the maternal allele is completely methylated among the two tested tissues. This maternal-specific methylation pattern was also maintained in the two types of the mutants with the paternal and maternal transmission of KO2, confirming the epigenetic stability of the main promoter of *Peg3*. Second, the U2 promoter was mostly methylated among the two tissues examined so far, displaying the methylation levels ranging from 70 to 91%. There was, however, a trend showing slightly higher levels of the DNA methylation of the U2 promoter in females than in males, but with no statistical significance in the samples of WT and KO2^(+/-p)^ (88.7 versus 80.3% with p = 0.1173 and 85.7 versus 83.2% with p = 0.3815 in brain, respectively). On the other hand, the methylation level of the U2 promoter was sexually different in the KO2^(-m/+)^ sample, showing a much lower level in females than in males (70 versus 90.8% with p = 0.0011 in brain). Males showed no significant methylation differences between WT and KO2^(+/-p)^ or KO2^(-m/+p)^ brains and tongue muscles. Interestingly, the brain DNA methylation of females indicated no significant differences between WT and KO2^(+/-p)^ (88.7 versus 85.7% with p = 0.3462), while WT and KO2^(-m/+)^ comparison indicated significantly lower methylation levels in KO2^(-m/+)^ (88.7 versus 70.0% with p = 0.0010). On the other hand, when comparing the tongue muscle DNA methylation of females between the WT and KO2^(+/-p)^ showed higher level of methylation for KO2^(+/-p)^ (85.5 versus 90.0% with p = 0.0034), while no significant differences were observed between WT and KO2^(-m/+)^ (85.5 versus 85.6% with p = 0.8865). Nevertheless, these observed changes in the DNA methylation levels of the U2 promoter may not be directly related to the actual activity of this promoter since the transcript driven by this promoter is almost undetectable in the brain (Fig 3B and S1A Fig). Overall, this series of DNA methylation analyses concluded that the main promoter is allele-specific regardless of the genotypes of the animals, whereas the U2 promoter is mostly methylated with at least 70% methylation level in the neonatal tissues. Yet, the DNA methylation level of the U2 promoter is sexually biased and, furthermore, sensitive to the genetic changes involving the main promoter of *Peg3*.

## Discussion

In the current study, we characterized the sexual, temporal and spatial specificity of the two promoters of *Peg3*, the main and U2 promoters. The results indicated that the main promoter is overall ubiquitous whereas the U2 promoter is stage and tissue-specific. Also, both promoters are sexually biased and, interestingly, are subject to dynamic fluctuations during late embryonic stages: the expression levels of *Peg3* in females tend to be higher close to birth but swiftly switches to a level lower than their male counterparts at birth. Overall, the promoters of *Peg3* display unusual sexually biased expression patterns and tissue-specific profiles.

**Fig 6:**
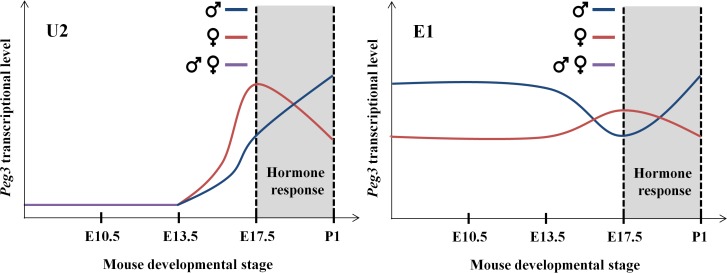
Interpretation of the sex and temporal specificity of the *Peg3* promoters. During early stages of embryogenesis, the expression levels of the alternative U2 and main promoters of *Peg3* are similar between male and females. However, the expression levels of both promoters in females become 2-fold higher than the levels in males at E17.5. The sexually biased expression levels further fluctuate at P1 with the levels in males becoming 2-fold higher than those in females. This fluctuation occurs at the developmental stage when sexual hormones reorganize various sexually dimorphic organs, such as brain. Thus, this hormone-driven reorganization is an untested hypothesis that is most likely responsible for the sexually biased expression levels observed from the *Peg3* locus.

The main and U2 promoters of *Peg3* both are sexually biased in terms of their expression levels (Fig 2). The expression levels of *Peg3* by the two promoters in females are 2-fold higher than the levels in males at the E17.5 stage, but this bias becomes opposite at the P1 stage with the levels in females becoming 2-fold lower than those in males (Fig 6). This is also consistent with the previous observation that the expression levels of *Peg3* in females are 2-fold lower than the levels in males at the P1 stage [12]. Furthermore, there have been several reports suggesting the presence of the sexual bias associated with the *Peg3* locus. For instance, mutagenesis experiments targeting mouse *Peg3* demonstrated that the loss-of-function-type mutations tend to affect more severely males than females [5,7]. In humans, the genomic region surrounding the promoter of *PEG3* is frequently hypermethylated in the patients of ovarian and breast cancers, revealing the epigenetic instability associated with human *PEG3* in female-specific organs [19–22]. Thus, these independent sets of observations again confirm the presence of the sexual bias associated with the mammalian *Peg3* locus. The observed sexual bias further suggests that the male and female mammals may require different gene dosages of *Peg3*. It is also important to note that the observed sexually bias is the more obvious during the late embryonic through early postnatal stages. This is particularly obvious for the U2 promoter since the expression levels by this promoter peak at this developmental stage. At the late embryonic stage, sexual hormones, such as testosterone, are known to rewire or reorganize the brain and other sexually dimorphic organs of mammals [23–25]. In humans, an external stimulus such as prenatal depression has shown an association to changes in PEG3 expression in the placenta [26]. Thus, it is likely that similar hormone-driven processes might be responsible for the fluctuating changes in the expression levels of *Peg3* between the two sexes (Fig 6). Overall, the results from the current study suggest that the *Peg3* locus may be subject to unknown mechanisms involving sexual differentiation in mammals.

During late embryonic stages, the expression levels driven by the main and U2 promoters are relatively high in muscle compared to the observed levels in brain (Fig 3). In particular, the expression levels by the U2 promoter were not even detectable at all in brain. The surveys on neonatal and adult tissues further confirmed that the U2 promoter has relatively little activity in the brains later in development although the main promoter becomes progressively activated in the neonatal and adult mouse brains (S1 Fig). This is quite unexpected at first, given the fact that previous studies have mainly focused on potential functions of *Peg3* in neuronal cells [6–9]. In hindsight, however, it is relevant to note that this gene was initially discovered through studies focusing on muscle development [10]. Recent comparative genomic studies also indicated that the 250-kb upstream region of mammalian *Peg3* is filled with many putative enhancers, and that many of these potential enhancers contain binding sites for MYOD, a master gene for muscle differentiation [15]. It is currently unknown how these potential enhancers are actually involved in the transcriptional regulation of the *Peg3* locus. Nevertheless, it is most likely that *Peg3* may play significant roles in muscle development and differentiation during embryogenesis.

The 4-kb genomic region surrounding the bi-directional promoter for *Peg3* and *Usp29* is an ICR (Imprinting Control Region) controlling the transcription and imprinting of the entire 500-kb imprinted domain [2,5]. According to the results from recent studies, the paternal deletion of this ICR causes domain-wide, global effects, including the abolition of the transcription of *Peg3* and *Usp29*, up-regulation of the surrounding genes, *Zim1* and *Zfp264*, and also bi-allelic expression of the maternally expressed *Zim2* [5]. In the case of the maternal transmission of KO2, no major effects were initially predicted given the silent and repressed state of the maternal allele of this ICR. Contrary to this prediction, however, the maternal deletion has been shown to cause the up-regulation of the remaining paternal allele of *Peg3* and *Usp29*. This unexpected outcome has been further confirmed through the higher survival rates and greater body weight of the mutants than those of the wild type littermates [5]. The current study also provides a set of similar results that the maternal deletion of the main promoter resulted in the up-regulation of *Peg3* and the U2 promoter (Fig 4). Since the U2 promoter is localized 26-kb upstream of the main promoter, the observed up-regulation may not be limited only to the bi-directional promoter of *Peg3* and *Usp29*. Thus, it is likely that the potential effects by the maternal deletion of the ICR may be domain-wide. This is further supported by the observation that DNA methylation levels of the U2 promoter is also affected in the pups with the maternal transmission (Fig 5). It is also possible that the DNA methylation levels of the remaining putative enhancers might be affected by the maternal deletion of this ICR, which will be interesting to pursue in the near future. Taken together, the results described above suggest that the transcription of the *Peg3* locus may be regulated through its multiple promoters that interact with each other.

## Materials and Methods

### Ethics Statement

All the mouse experiments were performed in accordance with National Institutes of Health guidelines for care and use of animals and also approved by the Louisiana State University Institutional Animal Care and Use Committee (IACUC), protocol #16–060.

### Mouse breeding experiments

The current study used the following two mouse strains: KO2 and DelKO [8,13]. Three-month-old male and female heterozygotes for these mutant alleles were bred together to derive two different sets of the pups: the first set with the paternal and maternal transmission of KO2 and DelKO, respectively, and the second set with the opposite transmission of the two mutant alleles. All the mice were housed at the DLAM (Division of Lab Animal Medicine) of LSU on a regular 12–12 dark-light cycle under a constant temperature 70°F and 50% humidity. All animals were given ad libitum access to water and Rodent Diet 5001. The nursing females were with Mouse Diet 5015. The mice were euthanized by CO2 asphixation in accordance with the rules and regulations set forth by the IACUC. The following primer sets were used for genotyping of the resulting progeny: for KO2, the deletion of exon 1 for *Peg3* and *Usp29* was detected using bac2082-F (5’- ACAACCCGGAGTTTTAGCAGAC -3’), bac6710-R (5’- GGATGTAAGATGGAGGCACTGT -3’), and bac2375-R (5’-AGGGGAGAACAGACTACAGA -3’); for DelKO, the deletion of exon 6 for *Peg3* was detected using Peg3-5arm (5’- CCCTCAGCAGAGCTGTTTCCTGCC -3’) and LoxR (5’- TGAACTGATGGCGAGCTCAGACC -3’), Peg3-5arm (5’- CCCTCAGCAGAGCTGTTTCCTGCC -3’) and Peg3-rev (5’- ACCCCATTCTCATCAGCTCCAGAG—3’); for sex determination, the Y chromosome was detected using mSry-F (5’-GTCCCGTGGTGAGAGGCACAAG-3’) and mSry-R (5’-GCAGCTCTACTCCAGTCTTGCC-3’). DNA was isolated from ear or tail snips after incubating the tissues at 65°C with the tail lysis buffer (50 mM Tris-Cl at pH 8.0, 100 mM EDTA at pH 8.0, 250 mM NaCl, 1% SDS, 20 μg/mL Proteinase K). PCR premix kit (Intron Biotech) was used for genotyping at the following conditions (step 1, 95°C-2 min; step 2, 95°C-30 sec, 60°C-30 sec, 72°C-60 sec for 33 cycles; step 3, 72°C-7 min). The information regarding individual primer sequences is available (S2 Table).

### RNA isolation, RT-PCR and qRT-PCR analysis

A commercial kit (Trizol reagent, Life technologies, cat: 15596018) was used for RNA isolation according to the manufacturer’s protocol. The total RNA was then reverse-transcribed using the M-MuLV reverse transcriptase (NEB, cat: M0253S). The cDNA was used as a template for RT-PCR (Maxime PCR Premix Kit, Intron Biotech) at the following conditions (step 1, 95°C-2 min; step 2, 95°C-30 sec, 60°C-30 sec, 72°C-60 sec for 33 cycles; step 3, 72°C-7 min). The U2 promoter and U0 exon were amplified using 36 cycles at step 2. The cDNA was also used as a template for quantitative PCR. This analysis was performed with iQ SYBR green supermix (Bio-Rad) using the ViiA*™* 7 Real-Time PCR System *(*Life Technologies). All qRT-PCR reactions were carried out for 40 cycles under standard PCR conditions with internal controls (*Gapdh* or *β-actin*). The experiments were performed in triplicates for each exon (*Peg3* E1, *Peg3* U2, *Peg3* U0, and *Usp29*). The ΔCt value was initially calculated by subtracting Ct value of a testing replicate of a given gene from the average Ct value of the internal control (*Gapdh* or *β-actin*). The fold difference for each replicate was then calculated by raising the ΔΔCt value as a power of 2 [27]. The relative expression levels of all samples were then calculated by dividing the calculated expression level of each sample by the expression level of the wild-type sample. The average and standard deviation for each sample triplicates were then calculated by compiling the normalized values. Each qRT-PCR result was confirmed using at least two independent experiments and is consistent with the data represented in the figures. The information regarding individual primer sequences is available in (S2 Table).

### DNA methylation analysis

DNA was first isolated from the brain and tongue muscle of one-day-old pups with the paternal and maternal transmission of KO2 allele as well as their wild-type littermates. The isolated DNA was subsequently treated with the bisulfite conversion protocol using the EZ DNA methylation kit (Zymo Research, cat: D5002). The converted DNA was used for PCR amplification targeting E1 and U2 (Maxime PCR Premix Kit, Intron Biotech). The following set of primers was used to amplify the U2 promoter: ECR4-Bis-a (5’- ATTGGTTTATAGTTAGGGAAGGAAGTAGT -3’) and ECR4-Bis-b (5'- AAATCTCTCTAAAACATAATACTATTCTAT -3'). The E1 promoter was amplified with the following set of primers: Peg3-met-11 (5’- AGAGGGTGTATGTTGTAGAGTAGTTAGGTG -3’) and Peg3-met-12 (5'- CATCCCTTCACACCCACATCCCATCC -3'). Each PCR product was further analyzed by restriction enzyme digestion-based COBRA (COmbined Bisulfite Restriction Assay) and the subsequent band densities were quantified using the ImageJ32 software (S1 Table) [18]. Detailed information regarding oligonucleotide sequences and restriction enzymes for COBRA is also available (S2 Table).

## Supporting Information

S1 Fig This file contains the additional results from the expression analyses of the promoters of *Peg3* and *Usp29* using the total RNA isolated from neonates and adult tissues. (TIF)

S2 Fig This file contains the additional results from the reciprocal cross of breeding experiments using two mouse strains, KO2 and DelKO. (TIF)

S3 Fig This file contains additional expression level analyses using qRT-PCR for the male neonate progeny resulting from the maternal transmission of KO2 (paternal transmission of DelKO). The paternal allele contribution was 4 to 2 times higher when comparing DelKO^(+/-p)^KO2^(-m/+)^ to KO^(-m/+)^ genotypes (p = 0.0215 for the U2 and p = 0.0001 for E1 promoters, respectively). The up-regulation of the paternal allele was shown by comparing KO2^(-m/+)^ to WT (p = 0.0223 for U2 and p = 0.0025 for E1 promotes, respectively.
(TIF)

S1 Table This table contains the additional information regarding the DNA methylation data analysis for the brain and muscle tissues of neonates. (XLSX)

S2 Table This table contains the information regarding the sequences of oligonucleotides used for RT-PCR and COBRA as well as the detailed conditions for individual PCR. (XLSX)
