# Supplementary figures and images for: Sex and Tissue Specificity of Peg3 Promoters
Source: PLoS One. 2016 Oct 6;11(10):e0164158. doi: 10.1371/journal.pone.0164158 (PMC5053485; doi:10.1371/journal.pone.0164158)

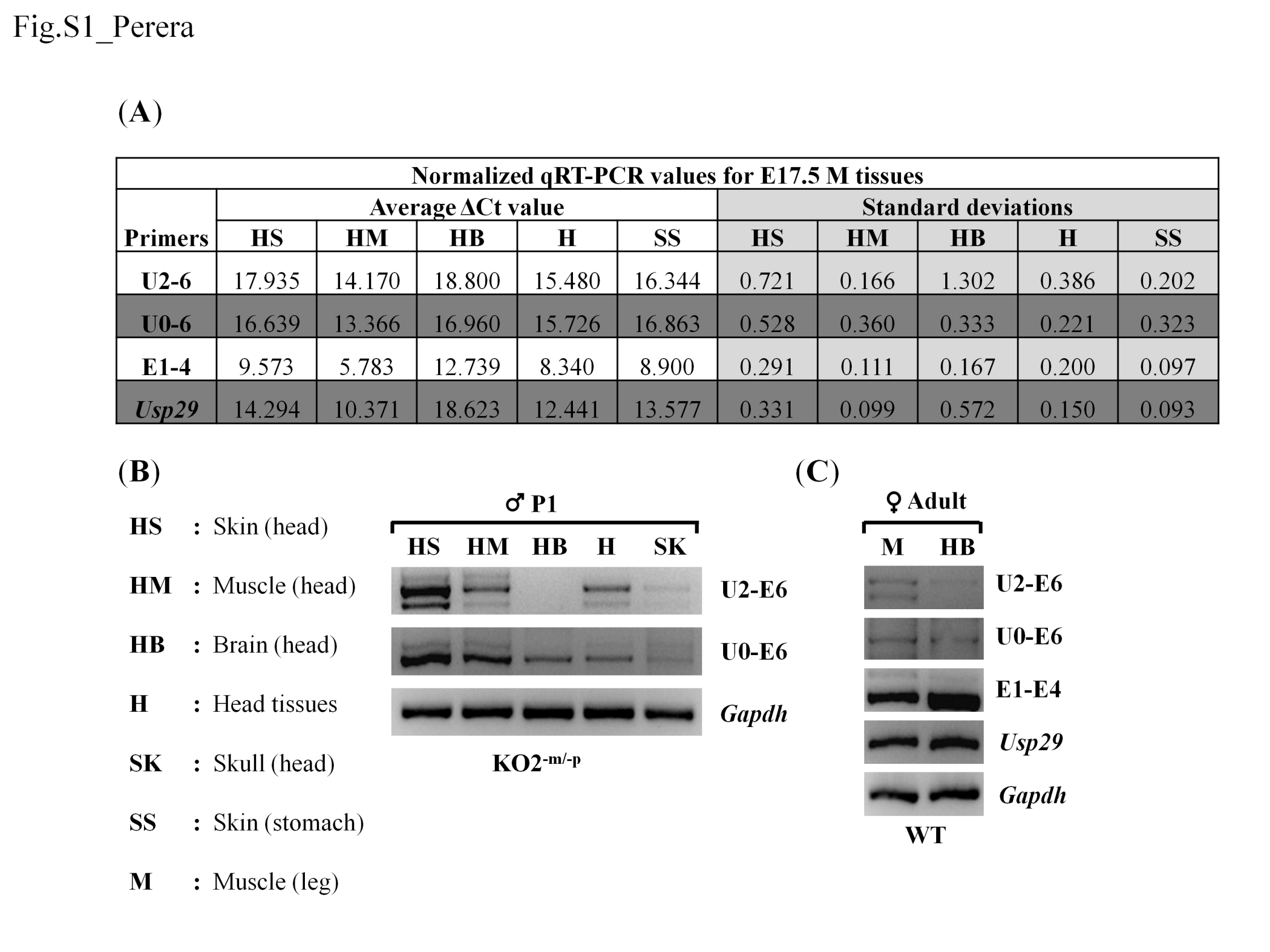

Supplement: S1 Fig — (TIF) [file pone.0164158.s001.TIF]

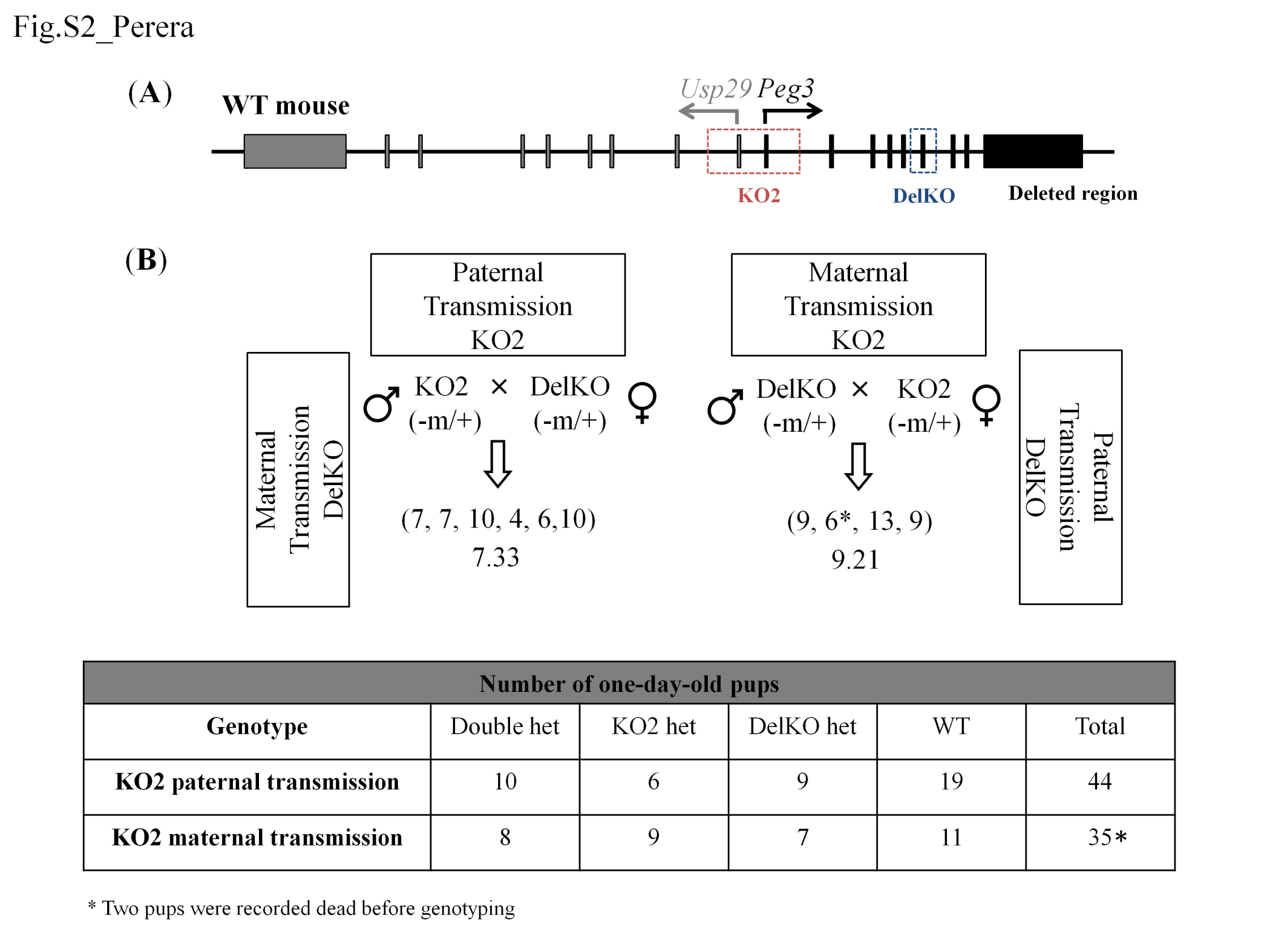

Supplement: S2 Fig — (TIF) [file pone.0164158.s002.TIF]

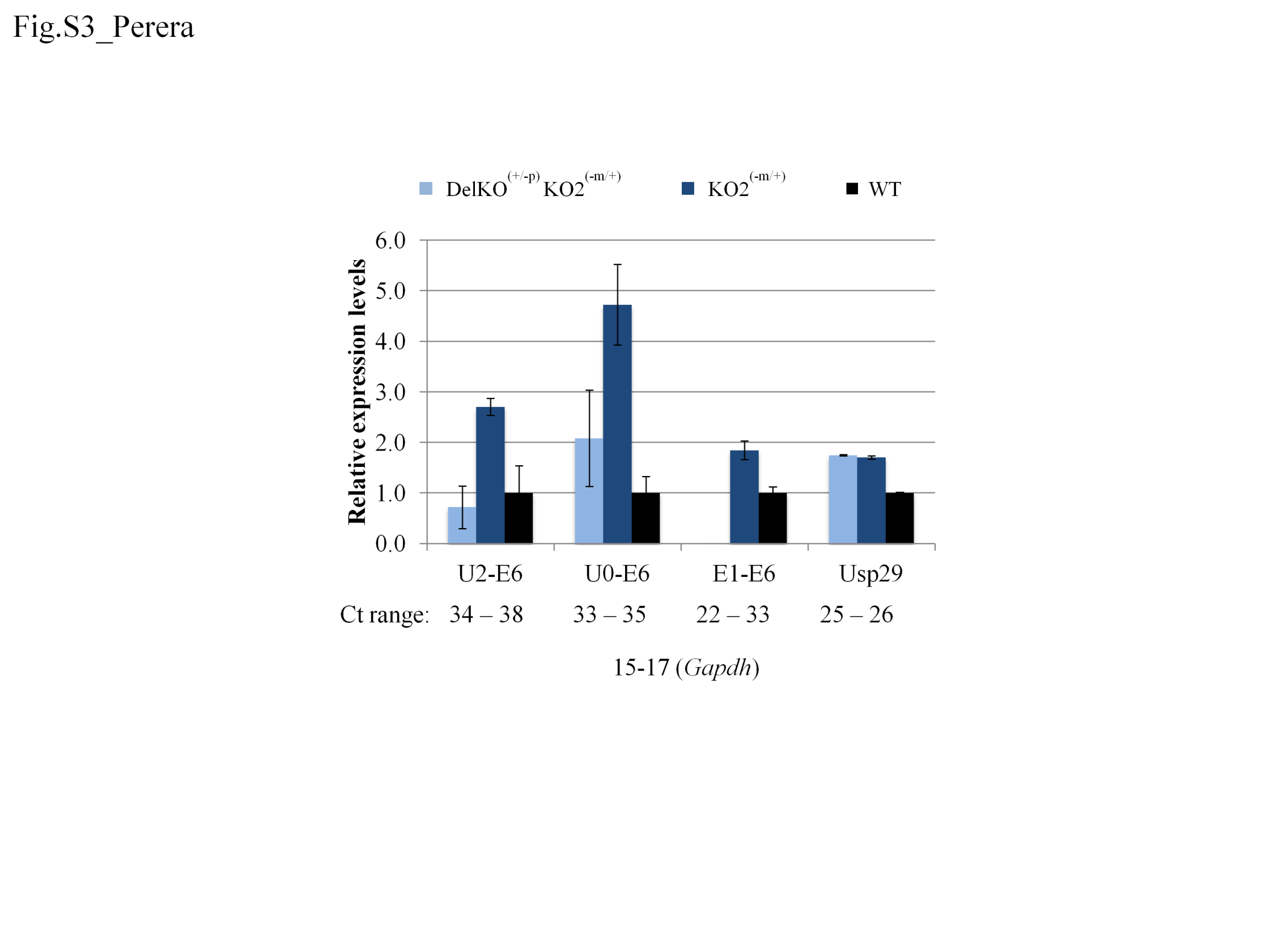

Supplement: S3 Fig — The paternal allele contribution was 4 to 2 times higher when comparing DelKO(+/-p)KO2(-m/+) to KO(-m/+) genotypes (p = 0.0215 for the U2 and p = 0.0001 for E1 promoters, respectively). The up-regulation of the paternal allele was shown by comparing KO2(-m/+) to WT (p = 0.0223 for U2 and p = 0.0025 for E1 promotes, respectively. (TIF) [file pone.0164158.s003.TIF]
